# Dynamic Changes in Neurexins' Alternative Splicing: Role of Rho-Associated Protein Kinases and Relevance to Memory Formation

**DOI:** 10.1371/journal.pone.0018579

**Published:** 2011-04-12

**Authors:** Gabriela Rozic, Zipora Lupowitz, Yael Piontkewitz, Nava Zisapel

**Affiliations:** 1 Department of Neurobiology, George S. Wise Faculty of Life Sciences, Tel-Aviv University, Tel-Aviv, Israel; 2 Department of Psychology, Faculty of Social Sciences, Tel-Aviv University, Tel-Aviv, Israel; Harvard University, United States of America

## Abstract

The three neurexins genes (NRXN1/2/3) encode polymorphic synaptic membrane proteins that are involved in cognitive functioning. Neurexins' selectivity of function is presumably conferred through differential use of 2 promoters and 5 alternative splicing sites (SS#1/2/3/4/5). In day-old rat brain neurons grown in culture, activation (depolarization) induces reversible, calcium dependent, repression of NRXN2α SS#3 insert. The effects of depolarization on NRXN1/2/3α splicing and biochemical pathways mediating them were further studied in these neurons. NRXN1/2/3α splicing in the course of memory formation *in vivo* was also explored, using fear conditioning paradigm in rats in which the animals were trained to associate an aversive stimulus (electrical shock) with a neutral context (a tone), resulting in the expression of fear responses to the neutral context.

In the cultured neurons depolarization induced, beside NRXN2α SS#3, repression of SS#3 and SS#4 exons in NRXN3α but not NRXN1α. The repressions were mediated by the calcium/protein kinase C/Rho-associated protein kinase (ROCK) pathway. Fear conditioning induced significant and transient repressions of the NRXN1/2/3α SS#4 exons in the rat hippocampus. ROCK inhibition prior to training attenuated the behavioral fear response, the NRXN1/2/3α splicing repressions and subsequent recovery and the levels of excitatory (PSD95) and inhibitory (gephyrin) synaptic proteins in the hippocampus. No such effects were observed in the prefrontal cortex. Significant correlations existed between the fear response and hippocampal NRXN3α and NRXN2α SS#4 inserts as well as PSD95 protein levels. Hippocampal NRXN1α SS#4 insert and gephyrin levels did not correlate with the behavioral response but were negatively correlated with each other.

These results show for the first time dynamic, experience related changes in NRXN1/2/3α alternative splicing in the rat brain and a role for ROCK in them. Specific neurexins' transcripts may be involved in synaptic remodeling occurring at an intermediate (hours) time scale in the course of memory formation.

## Introduction

Mammalian neurexins are highly polymorphic, primarily synaptic proteins that are transcribed from three unlinked neurexins genes, designated NRXN1, NRXN2 and NRXN3, each transcribed from 2 promoters to yield longer (α) and shorter (β) forms. All α-neurexins are variably spliced at five sites, referred to as SS# 1 to 5 whereas the shorter β-neurexins contain only SS#4 and 5 which may give rise to thousands of different transcripts [1], [2], [3]. Alterations in genes encoding neurexins have been implicated in diseases such as autism, schizophrenia and addiction [4].

Binding of neurexins to certain synaptic proteins (i.e. neuroligins, leucine-rich repeat transmembrane neuronal proteins (LRRTMs), GABA_A_ receptors and Cbln1/Glutamate receptor delta2 (GluRd2) form trans-synaptic complexes which regulate glutamatergic and GABA-ergic transmission and subsequently the excitatory/inhibitory balance in brain networks [5]–[10]. There is emerging evidence that certain neurexins polymorphs may differentially bind to such ligands and presumably affect their trans-synaptic properties. Whereas neuroligins bind neurexins containing or lacking SS#4 insert, LRRTMs 1 and 2 and GluRd2 interact specifically with α and β neurexins lacking an insert at SS#4 [5]. Alpha-neurexins containing the insert in SS#4 may mediate GABA-ergic synaptic protein recruitment and stabilization [10]. However, despite their apparent significance for maintaining the synaptic excitatory-inhibitory balance, very little is known about the dynamics of NRXN1/2/3 splicing patterns, mechanisms that regulate them and the functional outcomes of such.

Depolarization of neurons for 6 to 24 hours has been shown to affect splicing patterns of a number of genes encoding synaptic proteins [11]. We have recently observed that depolarization selectively repressed NRXN2α SS#3 splicing in primary cultures of cortical neurons leading to decrease in exon 11 (E11) included transcripts. E11 NRXN2α splicing repression was time dependent, reaching a maximum within 6 hours, and reversible, recovering baseline levels of E11-included transcripts within 6 hours after removal of the depolarizing medium. Cell viability was not affected under these conditions [12]. NRXN2α splicing repression was Ca ^2+^ dependent and completely abolished in the presence of BAPTA-AM, a chelating agent that lowers intracellular Ca ^2+^ [12].

Considering the role of NRXN1α and NRXN3α in mental health and cognitive functioning it was pertinent to ask whether neuronal activation would affect their splicing patterns as well. In the present study we investigated the alternative splicing of the two other neurexins in the depolarized neurons and found selective and reversible splicing repression at both SS#3 (E11) and SS#4 (E20) in NRXN3α but not in NRXN1α. The downstream pathways controlling E11 splicing repression in NRXN2α and NRXN3α and E20 splicing repression in NRXN3α were then explored using pharmacological agents which inhibit or activate specifics intracellular mechanisms, focusing on calcium regulated pathways. These experiments demonstrated the involvement of the calcium/protein kinase C (PKC)/Rho-associated protein kinase (ROCK) pathway in the splicing repression.

Emerging evidence link the ROCK pathway to the expression of anxiety- and fear-related behavior [13], [14]. Different studies have shown the involvement of the hippocampus and the prefrontal cortex in fear conditioning [15]. We therefore used fear conditioning paradigm to study potential experience dependent effects on NRXN1/2/3α expression and splicing in the course of memory formation in the rat brain, focusing on the hippocampus and prefrontal cortex. Since peripheral administration of the ROCK inhibitor hydroxyfasudil has been found to be effective in the brain *in vivo*, as evidenced by enhancement of working memory [16], we used this inhibitor to evaluate the role of ROCK in fear memory and the experience related changes in hippocampal NRXN1/2/3α SS#3 and SS#4 splicing. Hydroxyfasudil is a specific Rho-kinase inhibitor and has >100-fold greater selectivity to ROCK over other protein kinases both in vitro and in vivo; its inhibitory effect on ROCK is 100 times greater than on PKC and 1,000 times greater on myosin light-chain kinase (MLCK) [17]–[20]. To confirm the ability of this hydroxyfasudil to inhibit ROCK activity in vivo under the experimental conditions we measured the amount of phosphorylated cofilin, an endogenous ROCK substrate [21] in the hippocampal tissues.

There is significant evidence for cross-talk of neurexins and PSD-95, a family of excitatory postsynaptic scaffolding proteins found in excitatory synapses that controls the balance between excitatory and inhibitory synapses, and of gephyrin, a scaffolding protein found exclusively in inhibitory synapses in contacting dendrites [22]. Considering the role of SS#4 isoforms in the formation of neurexins' trans-synaptic complexes, the levels of excitatory (PSD95) and inhibitory (gephyrin) synaptic proteins were also assessed.

## Results

### Depolarization represses NRXN3α but not NRXN1α splicing in brain cortical neurons

**Figure 1:**
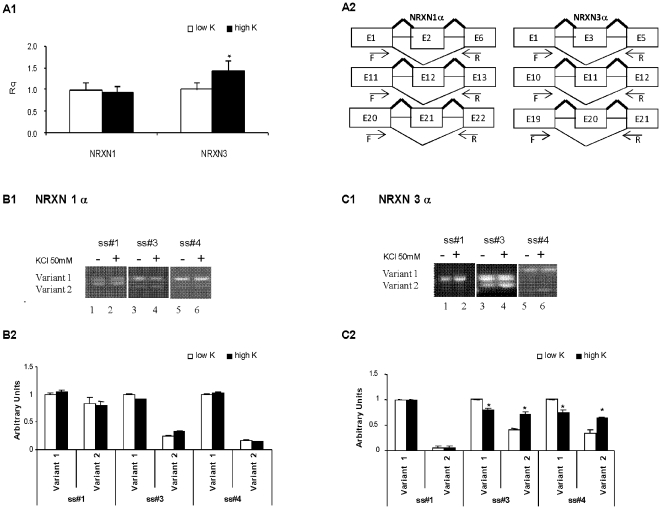
Depolarization differentially affects NRXN1α and NRXN3α splice variants expression. (A1) Real-time PCR analysis of total NRXN1α and NRXN3α transcripts 6 hours after treatment of primary cortical rat neurons with 5 (low K) and 50 mM (high K) KCl respective to GAPDH. (A2) Schematic representation of alternative splicing at SS#1, SS#3 and SS#4 of NRXN1α and NRXN3α. (B1, C1) Rat cortical neurons grown in culture for 14 days were incubated with 5 (lane 1, 3 and 5 low K) and 50 mM (lane 2, 4 and 6 high K) KCl for 6 hours. Relative quantities of exon-included (variant 1) and -skipped (variant 2) mRNA products were measured by RT-PCR analysis with primers designed to amplify across SS#1, SS#3 and SS#4. Representative results of NRXN1α (B1) and NRXN3α (C1) splice variants are shown in the gel panels. The bands were densitometrically quantified using the Image J program (B2-C2). The semi-quantitative estimation of the exon-included and the exon-skipped transcripts respective to the sum of both variants is depicted. (Values are mean +SEM from at least 3 experiments, * = P<0.05; t-test).

Cultured neurons from rat brain cortical areas expressed all expected NRXN1α and NRXN3α splice variants at splice sites SS#1, SS#2, SS#3 (the alternative exon E11 in NRXN3α and its homolog E12 in NRXN1α) and SS#4 (the alternative exon E20 in NRXN3α and its homolog E21 in NRXN1α) and the respective β forms at SS#4 [2]. NRXN3α and NRXN1α SS#3 and SS#4 exon-included transcripts were predominant under basal conditions (Figure 1).

Depolarization of the neurons (50 mM KCl, 6 hours) induced a significant increase in NRXN3α transcript levels whereas NRXN1α levels were unaffected (Figure 1 panel A1). There was no evidence for an effect of depolarization on NRXN1α alternative splicing (Figure 1 panels B1 and B2) but in NRXN3α exon preference at both SS#3 and SS#4 shifted towards exon exclusion (Figure 1 panels C1 and C2). Notably, splicing repression was associated with decrease in exon included and concomitant increase in exon excluded transcripts, relative to the total amount of transcripts.

**Figure 2:**
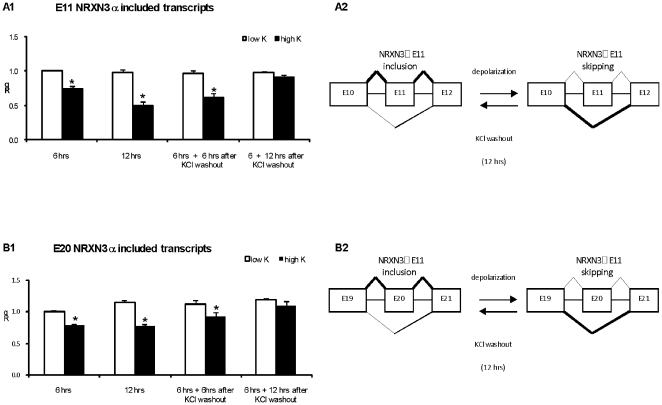
The depolarization-induced repression of E11 NRXN3α splicing is reversible. Real-time PCR quantification of (A1) E11 and (B1) E20 NRXN3α included transcripts expressed in rat cortical neurons incubated with 5 and 50 mM KCl for 6 hours. Reversibility of the induced depolarization effects on the expression of E11 and E20 NRXN3α included transcripts was monitored after 6 or 12 hours of depolarization and following an additional recovery period of 6 or 12 hours in culture medium after KCl washout. Rq values are mean +SEM from at least 3 experiments (* = P<0.05; t-test). (A2-B2) Schematic representation of the high KCl effects on E11 NRXN3α splicing.

The time course of NRXN3α E11 and E20 splicing repression was studied by applying the depolarizing medium (50 mM KCl) for 6 and 12 hours and exon included transcripts were measured using real time PCR. To examine the reversibility of this response the depolarizing medium was applied for 6 hours and then replaced with growth medium (5 mM KCl) and exon included transcripts were measured for up to 12 hours afterwards. As can be seen in Figure 2, E11 and E20 NRXN3α splicing repression reached a maximum within 12 hours. The splicing repression was reversible and baseline levels of the E11 and E20 NRXN3α included transcripts were attained within 12 hours of the recovery period.

### E11 and E20 NRXN3α splicing repression depends on RNA transcription but not *de novo* protein synthesis

**Figure 3:**
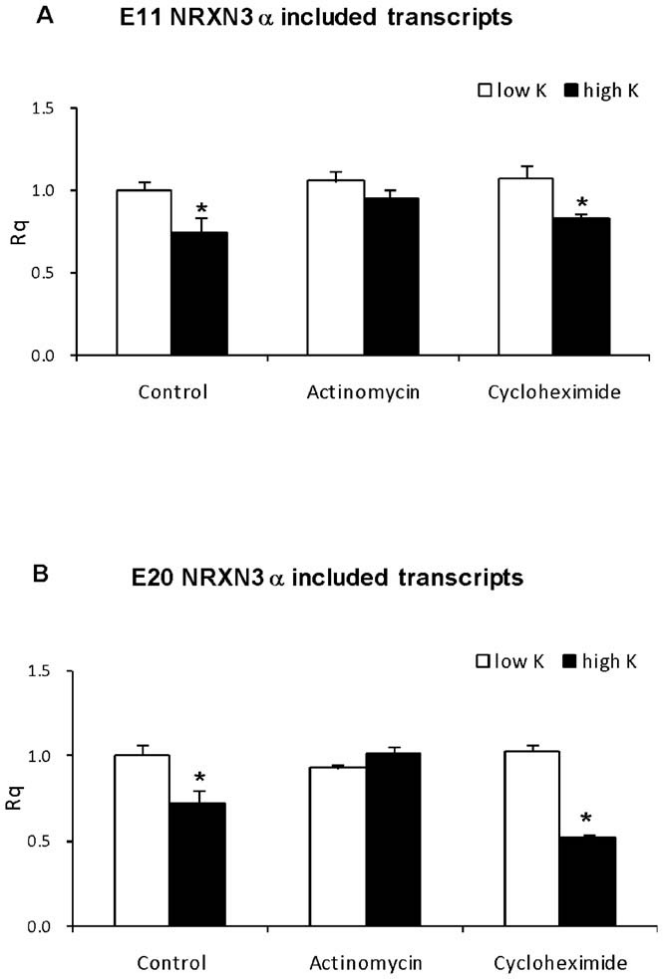
The depolarization-induced repression of NRXN3α E11 and E20 splicing depends on RNA synthesis but not protein synthesis. Rat cortical neurons grown in culture for 14 days were incubated with 5 and 50 mM KCl for 6 hours in the absence (control) or presence of actinomycin (10 ug/ml), cycloheximide (10 ug/ml). Quantification of NRXN3α (A) E11 and (B) E20 included transcripts was performed by Real-time PCR RT-PCR. Rq values are mean +SEM from at least 3 experiments (* = P<0.05; t-test).

The effects of the mRNA synthesis inhibitor actinomycin D and of the translational inhibitor cycloheximide on the expression of NRXN3α SS#3 and SS#4 splice variants were explored. As can be seen in Figure 3, actinomycin D did not affect exon 11 or exon 20 included transcripts at baseline but completely abolished the depolarization-induced decrease in both NRXN3α E11 and E20 included transcripts. Cycloheximide did not affect NRXN3α SS#3 transcripts at baseline or the depolarization-induced E11 and E20 splicing repressions (Figure 3A and B).

### The depolarization-induced E11 splicing repression is Ca^2+^ dependent and mediated via activation of PKC pathway

**Figure 4:**
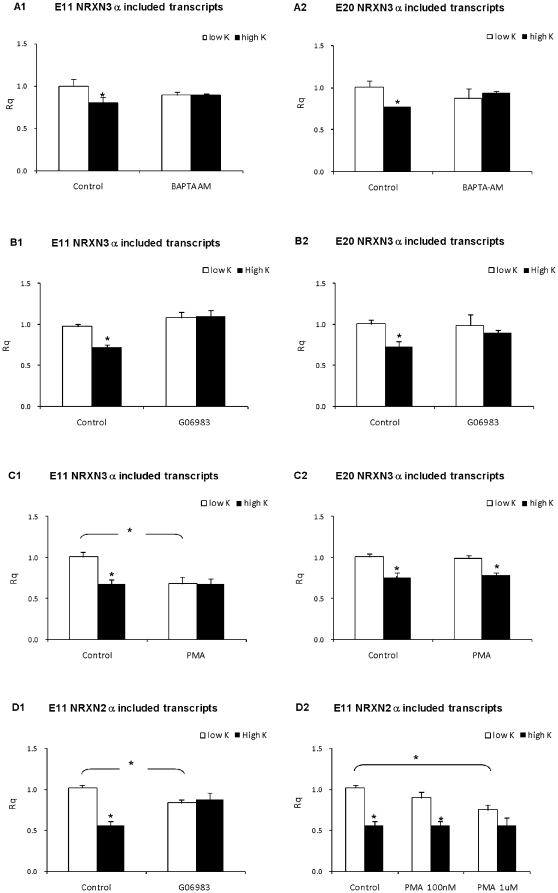
Involvement of Ca^2+^ and PKC in E11 and E20 splicing repression. Rat cortical neurons grown in culture for 14 days were incubated with 5 and 50 mM KCl for 6 hours in the absence or presence of BAPTA-AM (100 uM, panels A1 and A2), Gö6983 (1 uM; panels B1, B2, and D1) and PMA (100 nM, panels C1, C2 and D2). Real-time PCR analysis of E11 NRXN3α (panels A1, B1,and C1) E20 NRXN3α (panels A2, B2 and C2) and E11 NRXN2α (panels D1 and D2) included transcripts was performed. Rq values are mean +SEM from at least 3 experiments (* = P<0.01; t-test).

The role of Ca^2+^ in the depolarization-induced exclusion of E11 and E20 NRXN3α was explored using the cell-permeable intracellular Ca^2+^ chelating agent BAPTA-AM. As can be seen in Figure 4 panels A1 and A2, BAPTA-AM (100 uM) did not affect the amount of E11 and E20 NRXN3α included transcripts at baseline but completely abolished the depolarization-induced NRXN3α E11 and E20 splicing repression, as was the case with E11 NRXN2α splicing [12].

**Figure 5:**
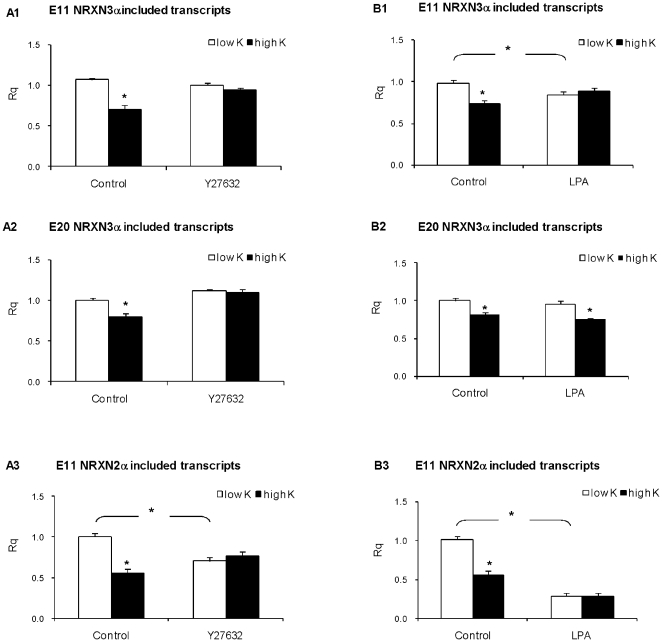
The depolarization-induced repression of E11 and E20 Splicing is mediated by ROCK II. Rat cortical neurons grown in culture for 14 days were incubated with 5 and 50 mM KCl for 6 hours in the absence or presence of Y27632 (10 uM, panels A1 A2 and A3) or LPA (60 uM, panels B1, B2 and B3) Real-time PCR analysis of E11 NRXN3α (panels A1 and B1), E20 NRXN3α (panels A2 and B2) and E11 NRXN2α (panels A3 and B3) included transcripts was performed. Rq values are mean +SEM from at least 3 experiments (* = P<0.01; t-test).

The involvement of downstream Ca^2+^ dependent pathways in the depolarization-induced changes in NRXN2α and NRXN3α E11 splicing was studied using pharmacological tools aimed at activating or inhibiting the mechanisms of interest (Figure 4, Figure 5). It was expected that inhibition of a critical component in the downstream pathway will inhibit the depolarization-induced splicing repression whereas activation of the pathway under basal conditions will cause depolarization-like splicing repression in the neurons. Incubation of primary cortical neurons for 6 hours with the PKC inhibitor Gö6983 did not affect E11 and E20 NRXN3α splicing and decreased the amount of E11 NRXN2α transcripts at baseline (Figure 4 panels B1, B2 and D1). Gö6983 prevented the depolarization-induced splicing repression of NRXN3α E11 and E20 as well as NRXN2α E11; in the presence of this inhibitor E11 and E20 including transcripts levels were comparable to those in non-depolarized neurons (Figure 4 panels B1, B2 and D1). Exposure of the cortical neurons to the ubiquitous PKC activator, phorbol 12-myristate 13-acetate (PMA;100 nM) selectively reduced NRXN3α E11 splicing under the basal conditions and obviated the effects of depolarization on the splicing of this exon (Figure 4 panel C1). However, under the same conditions PMA had no effect on NRXN3α E20 or NRXN2α splicing at baseline and did not prevent the depolarization induced repression of these exons (Figure 4 panels C1, C2 and D2).

### Role of Rho-associated kinase (ROCK II) in NRXN2α SS#3 and NRXN3α SS#3 and SS#4 splicing repression

The involvement of the ROCK II, a downstream pathway to PKC, in NRXN2α and NRXN3α splicing was explored (Figure 5). Under basal conditions the ROCK II inhibitor Y27632 (10 uM) did not affect NRXN3α E11 and E20 splicing and suppressed NRXN2α E11 splicing. Under the high KCl conditions Y27632 prevented the depolarization induced repressions of NRXN3α E11 and E20 as well as NRXN2α E11 splicing (Figure 5 panels A1, A2 and A3). The involvement of ROCK II in the depolarization-induced splicing NRXN2α SS#3 and NRXN3α SS#3 and SS#$ splicing was further explored by the use of lysophosphatidic acid (LPA), which activates intracellular signaling pathways of phospholipase C (PLC)/PKC, Ras/mitogen-activated protein (MAP)- kinase, and Rho/Rho-associated kinase [23]–[25]. LPA decreased the amounts of E11 including NRXN2α and NRXN3α transcripts under low KCl, similar to the effect of depolarization, and obviated the repression seen under depolarization (Figure 5 panels B1 and B3). However, LPA was not sufficient to promote E20 NRXN3α splicing repression under basal (low K) conditions, nor obviate the depolarization-induced repression of NRXN3α E20 splicing (Figure 5 panel B2).

### Fear conditioning leads to E11 and E20 NRXN3α splicing repression in the hippocampus

**Figure 6:**
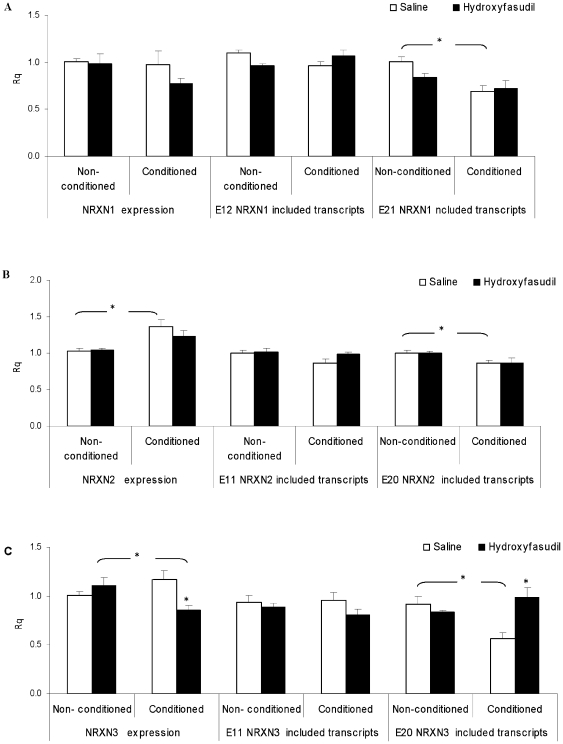
Fear conditioning-induced NRXN1/2/3α E20 splicing repression in the hippocampus. Rats were injected with Saline or with hydroxyfasudil (1 mg/kg) an hour prior to fear conditioning training. RNA samples from brain hippocampal area of conditioned and non-conditioned animal groups were extracted 2 hours after the training and tested by real time PCR. (A) NRXN1α (B) NRXN2α and (C) NRXN3α expression, E11 and E20 included transcripts levels are depicted. Rq values are mean +SEM of 7 animals/group in the non-conditioned 9 in the conditioned and 5 in the hydroxyfasudil treated groups (* = p<0.05 t test).

The effects of fear conditioning on NRXN1/2/3α splicing were studied in rats. We first compared NRXN1/2/3α expression and splicing in non-conditioned and conditioned animals that were injected i.p. with vehicle (saline) or hydroxyfasudil, an hour before the fear conditioning training. The animals were sacrificed 2 hours after the training and NRXN1/2/3α expression and alternative splicing patterns were subsequently assessed in the hippocampus and prefrontal cerebral cortex. No significant differences in NRXN1α and 3α expression were noted in the hippocampus in conditioned compared to non-conditioned animals treated with saline (Figure 6 panels A and C) but there was an increase in NRXN2α expression (, p = 0.017 Figure 6 panel B).

Fear conditioning resulted in significant changes in NRXN1/2/3α SS#4 splicing patterns. Thus, in the conditioned compared to non-conditioned animals there was a significant repression of SS#4 splicing (E20 or the respective E21 in NRXN1α) in NRXN1/2/3α (p = 0.002, p = 0.042 and p = 0.002, respectively) in the hippocampus Fig 6 panels A, B and C).

The effects of ROCK inhibition on NRXN splicing and memory formation in vivo were studied. A main problem with studies in vivo is the accessibility of the affected organ to the drug. Since peripheral administration of the ROCK inhibitor hydroxyfasudil has been found to be effective in the brain in vivo, we chose to use it for the in vivo experiment. Following administration of hydroxyfasudil, i.p., one hour before the training total NRXN3α expression tended to decrease (p = 0.051) in the conditioned animals only (Figure 6 panel C). In addition, the ROCK inhibitor attenuated the repression of NRXN1/2/3α SS#4 splicing in the hippocampus in the conditioned significantly compared to non-conditioned animals (Figure 6 panels A, B and C).

**Figure 7:**
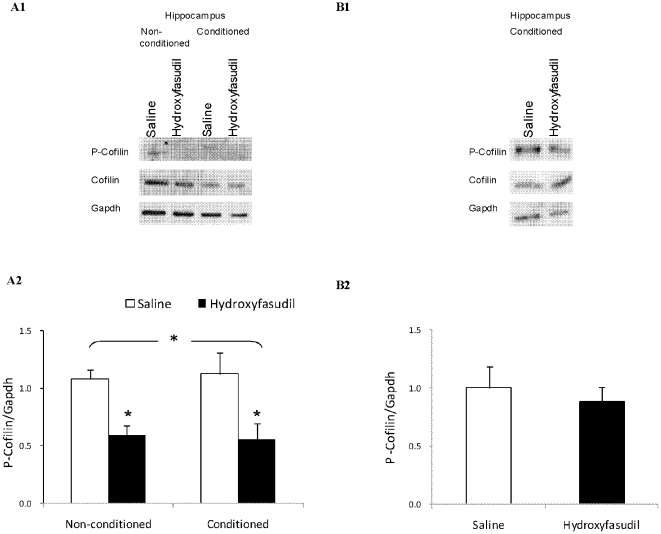
Peripheral Administration of Hydroxyfasudil reduces Phospho-Cofilin Levels in the Hippocampus. Rats were injected with Saline or with hydroxyfasudil (1 mg/kg) an hour prior to fear conditioning training. Conditioned and non-conditioned animals were sacrificed 2 hours after the training (panels A1 and B1) or 48 hours afterwards (panels B1 and B2) and the content of cofilin, phosphocofilin and gapdh were assessed by immunoblot in samples from the brain hippocampal area using specific antibodies. (A1-B1) Representative immunoblots of the proteins in the hippocampus. (A2-B2) The levels of phosphocofilin (P-Cofilin) relative to gapdh are depicted in panels A2 and B2. Values are mean +SEM, * = p<0.05 t test; n = 3 for the non-conditioned and n = 5 for the conditioned groups).

To evaluate the degree of inhibition of ROCK under the experimental conditions we assessed the amount of cofilin and phospho-cofilin in the hippocampi of non-conditioned and conditioned animals sacrificed 2 hours after training (Figure 7 panels A1 and A2). The amount of cofilin as compared to GAPDH was not affected by fear conditioning of hydroxyfasudil administration. However, a decrease of ∼50% in phospho-cofilin was observed following hydroxyfasudil administration in non-conditioned and even more in the conditioned animals (Figure 7 panel A2). The effect of hydroxyfasudil on phosphor-cofilin was no longer found in the conditioned animals sacrificed 48 hours later (Figure 7 panel B2).

Fear conditioning did not affect significantly NRXN1/2/3α SS#3 and SS#4 splicing in the prefrontal cortex compared to non-conditioned animals with or without hydroxyfasudil (data not shown).

### Fear conditioning leads to a ROCK independent increase in PSD95 levels in the hippocampus

**Figure 8:**
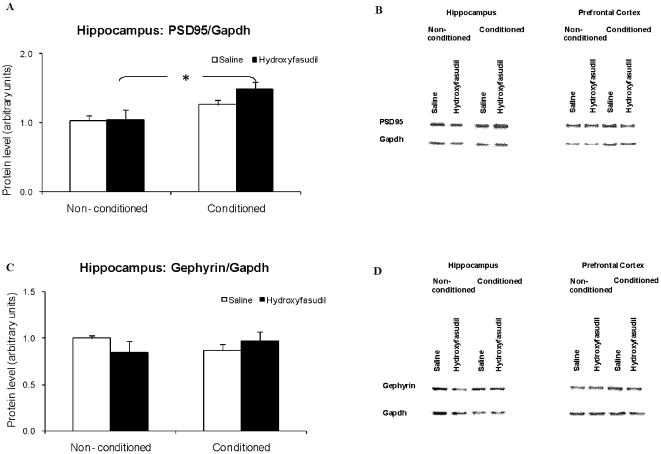
Fear conditioning effects on PSD95 and gephyrin levels in the hippocampus and prefrontal cortex. Hippocampus and Prefrontal Cortex protein samples from rats injected with saline or hydroxyfasudil (1 mg/kg) were extracted 2 hours following fear conditioning training and tested by immunoblotting. (A) PSD95 levels in the hippocampus; (B) Representative immunoblot of PSD95 in the proteins samples from the hippocampus and prefrontal cortex; (C) Gephyrin levels in the hippocampus; (D) Representative immunoblot of gephyrin in the proteins samples from the hippocampus and prefrontal cortex. Values are mean +SEM of 3 animals/group in the non-conditioned and 5 in the conditioned group (* = p<0.05 t test).

The effects of fear conditioning training on PSD95 and gephyrin levels were assessed at 2 hours after training (Figure 8). In the conditioned compared to non-conditioned animals PSD95 levels in the hippocampus tended to increase. The increase was significant in hydroxyfasudil treated animals. No changes were observed in gephyrin levels in the hippocampus at this time point. There were no significant changes in the levels of these proteins in the prefrontal cortex (Figure 8).

### Fear conditioning memory formation is associated with ROCK mediated NRXN3α E20 splicing in the hippocampus

**Figure 9:**
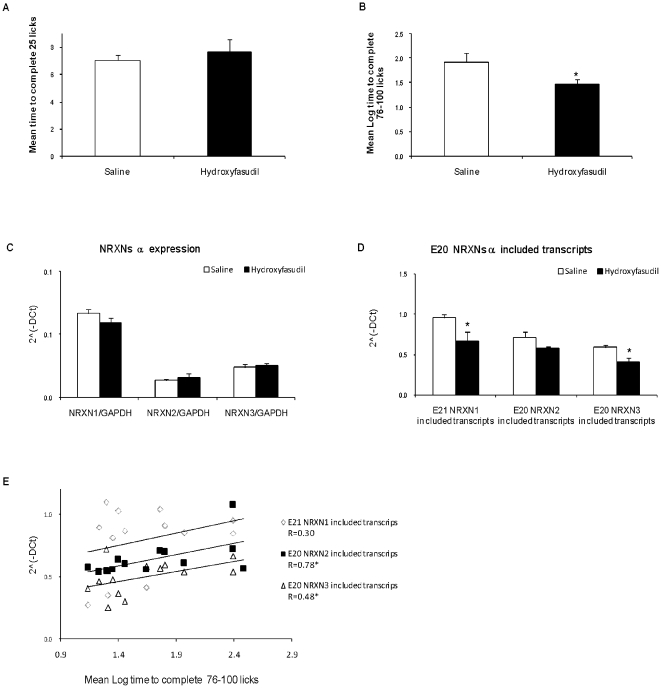
Fear conditioning is attenuated in hydroxyfasudil-treated rats. Rats were injected with hydroxyfasudil (1 mg/kg.) or saline 1 hour before fear conditioning. Memory performance was tested 48 hours afterwards and animals were sacrificed 2 hours later and NRXN1/2/3α expression and splicing assessed by Real Time PCR. (A) Mean±SEM times to complete 25 licks prior to tone onset. (B) Mean±SEM times (logarithmically transformed) to complete 25 licks in the presence of a tone that was previously paired with shock in saline and hydroxyfasudil-treated rats. Hydroxyfasudil reduced fear conditioning as manifested in shorter lick time after tone onset compared to controls. * = significant difference (p<0.05) between the treated and the control groups. (C) NRXN1/2/3α expression (D) NRXN1/2/3α E20/E21 transcript levels. (E) Significant correlations between the behavioral response and the amount of E21 NRXN1α and E20 NRXN2α and NRXN3α included transcripts in the hippocampus. Values are mean +SEM of 7 animals per group (*p<0.05).

The effects of peripheral administration of hydroxyfasudil (i.p. injection) 1 hour before fear conditioning on the behavioral response to tone assessed 48 hours later were assessed. The hydroxyfasudil and saline treated groups did not differ in the mean time needed to complete 51–75 licks prior to tone onset (tested 48 hours after training; p>0.5, Figure 9A), indicating that hydroxyfasudil administration had no effect on animals' unconditioned drinking. The behavioral response to tone (tested 48 hours after training) was significantly impaired in the animals who received hydroxyfasudil prior to the training, as reflected in the shorter times to complete 25 licks after tone onset (indicative of lower fear) in the hydroxyfasudil treated rats compared to rats that received saline (main treatment effect (F_(1,13)_ = 4.8, p<0.05; Figure 9B).

The administration of hydroxyfasudil before fear conditioning training had no effect on NRXN1/2/3α expression when measured two hours after test, but resulted in significantly lower splicing of E21 NRXN1α and E20 NRXN3α in the hippocampus as compared to vehicle treated animals (p = 0.038 and 0.007 respectively) and a tendency toward reduction in E20 NRXN2α p = 0.065; Figure 9C and D). No effects were observed in the prefrontal cortex. The behavioral fear response was significantly correlated with the amount of NRXN2α (p = 0.001) and NRXN3α (p = 0.048) E20 included transcripts in the hippocampus (measured at two hours after testing) but not with E21 NRXN1α included transcripts (Figure 9E).

### Fear conditioning memory formation is associated with ROCK mediated -regulation PSD95 and gephyrin in the hippocampus

**Figure 10:**
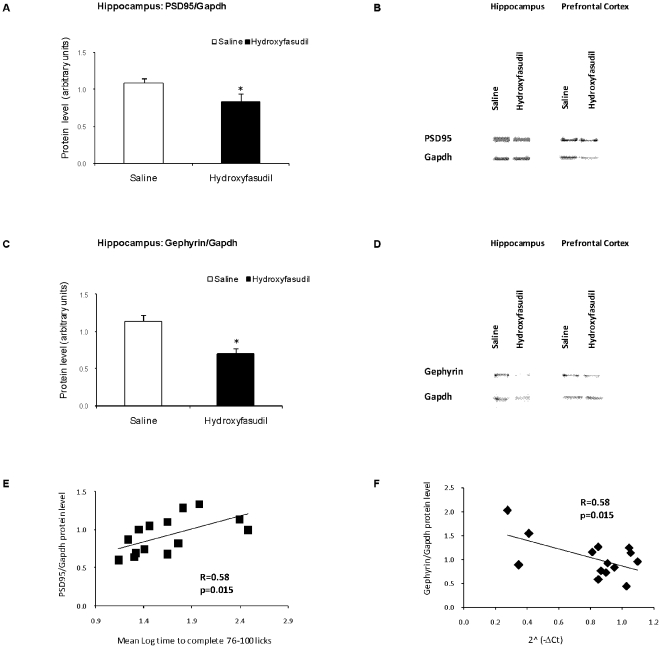
Fear Conditioning Effects on PSD95 and gephyrin levels in the hippocampus and prefrontal cortex. Rats were injected with hydroxyfasudil (1 mg/kg.) or vehicle 1 hour before fear conditioning training. Memory performance was tested 48 hours afterwards and animals were sacrificed 2 hours later. Protein samples were then prepared from the hippocampus and prefrontal cortex and assessed by immunoblotting. (A) PSD95 levels in the hippocampus; (B) Representative immunoblot of PSD95 in the proteins samples from the hippocampus and prefrontal cortex; (C) Gephyrin levels in the hippocampus; (D) Representative immunoblot of gephyrin in the proteins samples from the hippocampus and prefrontal cortex. (E) Significant correlations between the behavioral response and the amount of PSD95 (F) Significant correlations between E21 NRXN1α and the amount of gephyrin in the hippocampus. Values are Mean±SEM of 7 animals per group (*p<0.05).

Peripheral administration of hydroxyfasudil (i.p. injection) 1 hour before fear conditioning training resulted in significantly lower levels of PSD95 and gephyrin in the hippocampus measured at two hours after testing (i.e. 50 hours after training) as compared to conditioned animals that were treated with saline (Figure 10 A–D). Levels of these proteins in the prefrontal cortex were not affected (Figure 10 B, D). There was a highly significant correlation between the behavioral fear response and PSD95 levels in the hippocampus (p = 0.015; Figure 10 E). No such correlation was found for gephyrin. However, there was a significant negative correlation between NRXN1α SS#4 transcript levels and the amount of gephyrin in the hippocampus (p = 0.015; Figure 10 E).

## Discussion

Although neurexins have been known to play a role in the synaptic excitatory-inhibitory balance, very little is known about the dynamics of NRXN1/2/3α splicing patterns, mechanisms that regulate them and the functional outcomes of such. We have investigated the regulation of NRXN1/2/3α splicing in the context of neuronal depolarization and memory formation in adult animals. Our studies demonstrated specific and differential effects of depolarization on NRXN3α SS#3 and SS#4 splicing in neurons, and showed that splicing repression also occurred *in vivo* in the course of training. Notably, whenever there was a repression in the splicing, the alternative exon excluded transcript increased and the total amounts of transcripts was not reduced and even increased. Nevertheless, without further experiments on stability of certain transcripts, we cannot exclude the possibility that findings reported here may (at least in part) be due to alterations in the stability of individual transcripts.

Further investigations on the downstream pathways mediating the depolarization-induced changes in NRXN1/2/3α splicing revealed a major role of the Rho/ROCK pathway in mediating the Ca^2+^ dependent E11 NRXN2α and NRXN3α as well as E20 NRXN3α splicing repression. Some PKC isoforms are activated by calcium [26], [27]. PKC can activate ROCK [28]. The PKC inhibitor Gö6983 inhibited the depolarization induced E11 splicing repression. ROCK inhibition inhibited the depolarization-induced splicing repression. In principle, the PKC inhibitor Gö6983 may also directly inhibit voltage dependent Ca^2+^ channels [29] thus reducing Ca^2+^ entry and downstream responses. However, PKC activation by PMA provoked NRXN3α E11 splicing repression in the brain neurons similar to depolarization. PMA was however insufficient to provoke NRXN2α E11 NRXN3α E20 in the absence of depolarization.

The results further demonstrate a role of ROCK in mediating the Ca^2+^ dependent splicing repression at NRXN3α E11 and E20 and NRXN2α E11 *in vitro* and the experience related splicing of NRXN1/2/3α SS#4 exons and memory formation *in vivo*. Here, too, the ROCK inhibitor was able to block the depolarization dependent repression at all 3 splice sites. In principle, the ROCK inhibitor Y27632 may also directly inhibit PKC (Ki 26 uM). Therefore, the effects observed with this inhibitor might in principle reflect inhibition of PKC rather than ROCK. However, at 10 uM which is significantly below the Ki of this inhibitor for PKC, Y27632 is specific for the inhibition of ROCK [30]. Furthermore, activation of the PKC/ROCK pathway elicited splicing repression of E11 in both NRXN2α and NRXN3α but was not sufficient to repress NRXN3α E20 splicing in the absence of depolarization. A different ROCK inhibitor, hydroxyfasudil was used in vivo. This inhibitor that effectively inhibited ROCK in the hippocampus, as indicated by the decline in phospho-cofilin, also inhibited SS#4 exons splicing repression in the hippocampus as well as memory formation. The IC50 (concentration needed to attain 50% response) of hydroxyfasudil for ROCK is between 1–2 µM [18]. The injection of 1 mg/kg hydroxyfasudil resulted in a decrease of ∼50% in phospho-cofilin in non-conditioned and even more in the conditioned animals. Provided that cofilin is only phosphorylated via ROCK dependent pathways this would mean that the amount of hydroxyfasudil present in the hippocampus at 2 hours after administration is approximately equal to the IC50, namely 1–2 µM. At this concentration (and even 10 times higher than that), hydroxyfasudil is highly specific for ROCK and does not inhibit other potential kinases in the calcium regulated pathways such as PKC and MLCK [18]. However, as phosphorylation of cofilin is mediated by LIM kinases which are downstream of ROCK but can also be activated by other pathways, it is possible that the level of hydroxyfasudil in the hippocamapal cells was in fact higher than estimated. Therefore the possibility that additional pathways might have also contributed to the observed effects, cannot be excluded. The inhibition of the splicing repression by hydroxyfasudil is thus ROCK specific. It may thus be concluded that while the hydroxyfasudil-inhibited pathway is necessary and sufficient for E11 splicing repression, it is necessary but not sufficient to elicit the response at SS#4 in the absence of stimulation.

There is a great deal of parallelism between the Rho/ROCK pathway and the neurexins system. Both are involved in exocytosis, neuronal differentiation during development cognitive performances and mental retardation [31]–[41]. Our observation on ROCK-dependent changes in neurexins splicing in the course of memory formation may thus shed light on this apparent parallelism between ROCK activation, the neurexins system and memory formation.

Despite high structural similarities of the respective splice sites, both *in vitro* and *in vivo* experiments have demonstrated differential splicing patterns of the 3 NRXN1/2/3α in response to activation. Whereas depolarization of brain neurons resulted in SS#3 splicing repression in NRXN2α [12] and NRXN3α, SS#3 splicing in NRXN1α was not affected. Unlike brain neurons *in vitro* where activation resulted in suppression of SS#4 splicing exclusively at NRXN3α, *in vivo* there was a repression of SS#4 splicing in all three NRXN1/2/3α transcripts in the hippocampus after the training. A possible explanation for the differences in the results from cultured neurons and intact brain is that NRXN1/2/3α SS#4 splicing repression *in vivo* requires the involvement of additional cell types (glia, astrocytes, etc) or extracellular factors that are absent in the neuronal culture. It is also possible, taking into consideration that the splicing repression is reversible and has different kinetics for different splice sites in the different neurexins, that the picture observed at a certain time point reflects the temporary state. These options remain to be investigated.

The onset and recovery rates of the splicing repression also differ between the neurexins and was faster for NRXN2α SS#3 (E11) (6 hours; [12]) than NRXN3α (12 hours). Results *in vivo* are essentially compatible with a dynamic response. Thus, the experience related suppression in SS#4 splicing (in all three NRXN1/2/3α transcripts) was observed at 2 hours after the training and apparently reversed later on, as found at 2 hours after the testing. The differential effects of activation on SS#3 and SS#4 splicing in the 3 NRXN1/2/3α and the differences in respective onset and recovery rates strongly suggest a role of more than one factor, beside ROCK, in the alternative splicing of the various NRXN1/2/3α gene products.

The dynamics of the splicing repressions is compatible with a role of the particular transcripts in intermediate steps in synaptic remodeling. It is interesting to note that while NRXN1/2/3α SS#4 splicing was repressed after training it was the reversal of this process (i.e., the recovery in NRXN2α and NRXN3α E20 exon-included transcripts in the hippocampus) that correlated with the behavioral fear response. One explanation for the transient nature of the splicing repression is transfer of the memory traces from the hippocampus to other brain networks for memory consolidation. For hippocampus-dependent tasks, memory storage occurs first in the hippocampus and over time is transferred to cortical areas (for review, see [42]). We did not observe differences in NRXN1/2/3α expression and splicing in the prefrontal cortex in the conditioned animals but cannot rule out this possibility because such changes might be limited to certain areas or may not be involved in the long term memory consolidation.

One may argue that the reversal of the splicing repression is related to memory retrieval in the behavioral testing. Retrieval triggers a time interval when the original memory becomes labile or extinct [43]. However, the significant correlation between the spliced NRXN2α and NRXN3α SS#4 transcripts and behavior suggest that the reversal was part of the formation rather than extinction of the memory. Furthermore, the levels of PSD95 in the hippocampus were reported positively correlated with the behavioral fear response. Elevated levels of PSD-95 alter the ratio of excitatory to inhibitory synaptic contacts by sequestering members of the neuroligins family to excitatory synapses [44]. Considering the role of SS#4 in the differential binding of NRXN1/2/3 to neuroligins, LRRTM2 and Cbln1- GluRd2, it is possible that the changes in NRXN1/2/3α SS#4 splicing during fear conditioning promoted changes in trans-synaptic interactions of the neurexins polymorphs, and subsequently in glutamate and GABA-ergic transmissions, leading to memory formation. Specific neurexins' transcripts may thus be involved in synaptic remodeling occurring at an intermediate (hours) time scale in the course of memory formation.

On the other hand, lower gephyrin levels were inversely correlated with NRXN1α splicing and neither correlated with behavioral fear response. Previous studies have demonstrated that gephyrin mRNA and protein levels are upregulated after extinction training [45], [46]. Alpha-neurexins promote clustering of the gephyrin, as well as GABA_A_ receptor and neuroligin 2 at GABA-ergic synapses in a LNS6 domain dependent mode (where E20 is localized) [22]. It cannot be rules out that activation of ROCK in the course of fear conditioning training elicits the change in SS#4 NRXN1α splicing and the reversal of this effect subsequent changes in gephyrin levels are triggered by the testing and linked to the memory retrieval process.

To our knowledge this is the first demonstration of dynamic, experience related changes in NRXN1/2/3α alternative splicing in the course of memory formation. In this respect the data provide a new insight into the normal processing of memory traces by showing that the behavioral response is associated with an initial suppression but also requires subsequent recovery of SS#4 splicing. It remains to be further examined whether the splicing suppression results in loss or decrease in certain trans-synaptic complexes, to allow subsequent remodeling.

## Materials and Methods

### Materials

Minimal essential medium (MEM), Dulbecco's modification of Earle's medium (DMEM), heat-inactivated horse serum (IHS), L-glutamine, penicillin-streptomycin and EZ-RNA Total RNA Isolation Kit were purchased from Biological Industries (BI, Beit Haemek, Israel), B-27 supplement from Invitrogen (Carlsbad, CA), Verso™ RT-PCR System from Thermo Scientific, Taq DNA polymerase from Bioline (Luckenwalde, Germany) and KAPA SYBER FAST qPCR Kit from KapaBiosystems (Woburn, MA). All other inhibitors and activators were purchased from Sigma (Sigma-Aldrich, St. Louis, MO). Anti PSD95, gephyrin and gapdh antibodies were from Abcam, Cambridge, MA) and anti cofilin and phospho-cofilin antibodies from Cell Signaling Technology (Danvers MA).

### Animals

All experimental protocols conformed to the animal care guidelines of the National Institutes of Health (NIH) and approved by the Institutional Animal Care and Use Committee (animal welfare approval numbers A5010-01 and L-05-032).

### Cell culture and treatment

Cultures were prepared from postnatal day 1 Sprague-Dawley rats. Cortical tissue was digested with papain (100 U; 20 min) triturated to a single-cell suspension. Cells were plated onto 6 wells plates (2×10^6^ cells/plate) precoated with 100 µg/ml poly-L-lysine in MEM containing 5% IHS, 2% B-27 neuronal supplement, and 100 IU/ml of penicillin, 100 µg/ml of streptomycin, 2 mM glutamine and 6 mg/ml D-Glucose. Cells grown for 11–13 days in culture were incubated with 50 (“high K”) or 5 mM (“low K”) KCl in growth medium at 37°C in humidified atmosphere with 5% CO_2_ for 6 hours (found in optimization studies to ensure a fully developed response). RNA was extracted using EZ-RNA Total RNA Isolation Kit.

### Fear conditioning and drug injection

Adult (350–400 g) male Wistar rats were housed 3–4 to a cage under reversed cycle lighting (lights on: 1900–0700 h) with ad lib food and water, unless otherwise described. Fear conditioning was assessed in a thirst-motivated conditioned emotional response (CER) procedure, conducted in standard rodent test chambers equipped with a retractable bottle and a drinkometer (Campden Instruments, United Kingdom). The experimental protocol conformed to the guidelines of the Institutional Animal Care and Use Committee of Tel Aviv University, Israel, and to the guidelines of the NIH (animal welfare assurance number A5010-01, expires on 9.30.2011).

Thirty two adult (350–400 g) male Wistar rats participated in the experiment. They were housed 3–4 per cage under reversed cycle lighting (lights on: 1900–0700 h) with ad lib food and water. A 22 h water restriction schedule was initiated 10 days prior to the beginning of the experimental procedure. Handling (2 min/day) was initiated simultaneously with water restriction and continued for 5 days. On the next 5 days, rats were trained to drink in the experimental chamber for 20 min/day. Water in the test apparatus was given in addition to the daily ration of 2 hours given in the home cages. Fear conditioning procedure was conducted on days 11–14 as follows: On day 11, with the bottle removed, all animals were given 30 min habituation to the apparatus. Conditioning and drug injection were conducted 24 h later. One hour prior to the beginning of conditioning 16 rats received a single intraperitoneal (i.p.) injection of hydroxyfasudil (Hydroxyfasudil; 1.0 mg/kg in saline; Sigma, St Louis USA), and 16 rats) received an equivalent volume of saline (Saline). With the bottle removed, each rat was put into the chambers for 20 min. Thirteen rats from each drug condition received fear conditioning, consisting of 2 tone (10 s, 80 dB, 2.8 kHz)-shock (0.5 mA, 1 s) pairings, with shock immediately following tone termination, given 5 min apart (conditioned). The first pairing was given 5 min after placement in the box and rats were taken out 5 min after the second pairing. The 3 remaining rats from each drug condition rats were merely confined to the chamber for an identical period of time (non-conditioned). Two hours after the conditioning stage, the 6 non-conditioned (3 Saline and 3 Hydroxyfasudil), and 10 conditioned (5 Saline and 5 Hydroxyfasudil) rats, were sacrificed. The remaining 16 conditioned rats (8 Saline and 8 Hydroxyfasudil) were tested 48 hours later to assess their level of fear to the tone. Each rat was placed in the chamber with an access to the bottle. When the rat completed 75 licks, the tone was presented. Times to complete 25 licks prior to tone onset (licks 51–75) and after tone onset (licks 76–100) were recorded. Each rat was sacrificed 2 hours after the test session.

### Tissue dissecting, RNA and protein extraction

The brains were rapidly removed, frozen on dry ice and kept at −70°C. Tissue was then homogenizing and the RNA and proteins extracted using the NucleoSpin**®** RNA/Protein kit (Macherey-Nagel, GmbH & Co KG, Düren, Germany).

### RNA extraction and reverse transcription

Total RNA (1.5 µg) was denatured in the presence of a specific primer or oligo-dT for 10 min at 70°C. cDNA was then prepared (1 h at 42°C) using Verso™ RT-PCR System from Thermo Scientific. Reactions were blocked by 3 minutes incubation at 95°C and samples stored at −20°C.

### PCR amplification and DNA sequencing

cDNA sample aliquots were added to reaction mixtures containing 1.5 mM MgCl_2_, 200 µM dNTP, 500 nM of each primer and 1 U *Taq* DNA polymerase. Amplification reactions (PTC-200 thermal cycler) started with denaturation phase (3 min at 94°C) followed by repeated cycles of incubations (30 s at 94°C, 30 s at 62°C and 30 s at 72°C). PCR products were subjected to electrophoresis on 2.5% agarose gel and stained with ethidium bromide [10 µg/ml]. Gels were photographed on top of a 280 nm UV light box and images densitometrically quantified with the PC Image J program. RT-PCR analysis was performed with primers designed to amplify across splice sites SS#1, 3 and 4 in NRXN1α and NRXN3α as shown in Table 1. RT-PCR values are presented as a ratio of the NRXN1α or 3α splice variant's signal in the selected linear amplification cycle divided by the total NRXN1α or 3α signal (sum of all variants) respectively. The resultant value was expressed as arbitrary units. To compare between different experiments, these values were calculated as % of the respective value for the exon included splice variant in control cells treated with low K.

**Table 1:** Primers used in RT-PCR analysis.

Name	Primer (5′ to 3′)
NRXN1α SS#1 forward	5′-cagggacatgacggtgttcagt
SS#1 reverse	5′-TCAAAGGCCCCTGATCCCAAA
NRXN1α SS#3 forward	5′-gcagatacccttcgactgga
SS#3 reverse	5′-CCACACGTACTGTGTGCCATTC
NRXN1α SS#4 forward	5′-tcaaggcgtgtgcttgcagcaa
SS#4 reverse	5′-ACTTCACCGACCAGCCTCACAT
NRXN3α SS#1 forward	5′-atggtcatcccacctgtgac
SS#1 reverse	5′- TGATTTCACTGCTGCTGCTC
NRXN3α SS#3 forward	5′- gactacctccagggattctgc
SS#3 reverse	5′-CCACATCATCGTCCACTGTT
NRXN3α SS#4 forward	5′- caacccttcaggtggacaac
SS#4 reverse	5′-TTTCCTCCGATGGCTATTTG

### Real Time PCR analysis

The primers used for real time PCR analysis are depicted in Table 2. Gene expression values for the various NRXN1/2/3α and splice variants were calculated based on the comparative threshold cycle (Ct) method [47]. Ct values were corrected for 100% efficiency as described [48] according to the following formula: Ct _E = 100%_ = Ct _E_ { log (1+E)/log (2)}; where E = 10 ^-1/slope^.

**Table 2:** Primers used in Real Time PCR analysis.

Name	Primer (5′ to 3′)
NRXN1α exon 12 forward	5′-caggctatctcggcagg
exon 13 reverse	5′-GCTTGTTGATCATCCACG
NRXN2α exon 10 forward	5′-gtccctgcgattcatgtccc
exon 11 reverse	5′-CAGCCGACGCGCAGG
NRXN3α exon 11 forward	5′-ctgtatcaggataaactgtaactcc
exon 12 reverse	5′-CCACATCATCGTCCACTGTT
NRXN1α exon 14 forward	5′-cattgcaagcctacacttctatgc
exon 15 reverse	5′-AGGTTAGCACCATTTCCCAAGTC
NRXN2α exon 14 forward	5′-tccagggacccaggcaac
exon 15 reverse	5′-CTTGCTCAGGCCACCGATG
NRXN3α exon 14 forward	5′-actcgggacaacagtaatacccac
exon 15 reverse	5′-CTGGGCTAAGCCAGCCATATAG
NRXN1α exon 16 forward	5′-tcaaggcgtgtgcttgcagcaa
exon 21 reverse	5′-CGCTGTCTAGCAATCGCCAG
NRXN2α exon 16 forward	5′- gccctgtctgcaatgac
exon 20 reverse	5′-GCGCTCGTTATCAAAGTTC
NRXN3α exon 16 forward	5′- ctggaaaccagtgcaatgacc
exon 20 reverse	5′-GGCGTTCATTATCAGTGTTGC
GAPDH forward	5′-gacaactttggcatcgtgga
reverse	5′-ATGCAGGGATGATGTTCTGG

The corrected Ct data for a specific NRXN isoform, total NRXN mRNA and the housekeeping gene GAPDH mRNA in each sample were used to create ΔCt values for total NRXN in sample (Ct _total NRXN_ – Ct _GAPDH_) and specific isoform transcripts (Ct _specific NRXN isoform_ – Ct _total NRXN_). Thereafter, ΔΔCt values were calculated by subtracting the ΔCt of the untreated control sample from the Ct value of treated sample. Rq = 2^−ΔΔCt^.

### Immunoblotting

The protein samples (25 µg) were subjected to SDS-PAGE and immunoblotting. Blots were blocked with blocking buffer (5% non-fat dry milk in PBS or 0.1% BSA) for 1 hour at room temperature. Blots were subjected to the primary antibody (PSD95 1∶500, gephyrin 1∶500, gapdh 1∶1000 cofilin 1∶700, phospho-cofilin 1∶700) in PBS with 0.1% Tween 20 (PBST) overnight at 4°C. Blots were washed with PBST (4×10 min each) at room temperature on a bench top shaker. Secondary antibodies conjugated to IRDye 800 or IRDye 680 (Li-cor) were diluted 1∶10000 in PBST. The membranes were incubated with the fluorescently-labeled secondary antibodies for 1 hour at room temperature in the dark on a bench top shaker. The membranes were washed in PBST (4×10 min each) at room temperature in the dark on a bench top shaker and then briefly rinsed in PBS. Membranes were scanned and analyzed using an Odyssey IR scanner using Odyssey imaging software 3.0. (Li-cor). Scan settings were medium or high image quality, 160 µm resolution and intensity 3.0–5.0 for both channels. Antibody signals were analyzed as integrated intensities of regions defined around the band of interest in either channel.
